# Generation of a *scFv* Derived from an IgM-Producing Hybridoma for the Detection of REST Expression in Premalignant Lesions and Invasive Squamous Cell Carcinoma

**DOI:** 10.3390/ijms262411946

**Published:** 2025-12-11

**Authors:** Cynthia Rodríguez-Nava, Karen Cortés-Sarabia, Lidia Riaño-Umbarila, Baltazar Becerril-Luján, Yolanda Medina-Flores, Olga Mata-Ruíz, Lourdes Lloret-Sánchez, Berenice Illades-Aguiar, Luz del Carmen Alarcón-Romero, Carlos Ortuño-Pineda

**Affiliations:** 1 Laboratorio de Investigación en Citopatología e Histoquímica, Universidad Autónoma de Guerrero, Chilpancingo 39086, Mexico; 2 Laboratorio de Investigación en Inmunobiología y Diagnóstico Molecular, Universidad Autónoma de Guerrero, Chilpancingo 39086, Mexico; 3 Investigadora por México, Secretaría de Ciencia, Humanidades, Tecnología e Innovación, Cuernavaca 62210, Mexico; 4 Departamento de Medicina Molecular y Bioprocesos, Instituto de Biotecnología, Universidad Nacional Autónoma de México, Cuernavaca 62210, Mexico; 5 Laboratorio de Anticuerpos Monoclonales, Departamento de Biología Molecular y Validación de Técnicas, Instituto de Diagnóstico y Referencia Epidemiológico “Dr. Manuel Martínez Báez” (InDRE), Mexico City 01480, Mexico; 6 Laboratorio de Investigación en Biomedicina Molecular, Universidad Autónoma de Guerrero, Chilpancingo 39086, Mexico; 7 Laboratorio de Ácidos Nucleicos y Proteínas, Universidad Autónoma de Guerrero, Chilpancingo 39086, Mexico

**Keywords:** Cervical cancer, immunocytochemistry, *scFv*, REST

## Abstract

Cervical cancer (CC) can be prevented through continuous screening and the timely detection of cervical intraepithelial neoplasia (CIN) using immunohistochemistry techniques to identify biomarker expressions. In a previous study, we proposed nuclear REST loss as a biomarker in precancerous lesions and CC; however, no validated antibodies are available for detecting REST in cytology or cervical tissues. Although we have developed an IgM-type anti-REST monoclonal antibody capable of detecting REST in liquid-based cytology cells, it was not useful for the detection of REST in cervical tissues by immunohistochemistry. The main objective of this study is to generate single-chain variable fragments (*scFvs*) for the clinical evaluation of REST in cervical tissues from women with CIN and CC. Using RNA from an IgM-producing hybridoma anti-REST, we conducted RT-PCR and PCR to obtain the coding sequences for the variable regions of the heavy and light chains. These sequences were joined with a linker to create a single-chain antibody. The *scFv* was then cloned into the *pSyn1* vector, expressed in *E. coli* TG1, and purified through chromatography. Subsequently, it was characterized using immunological methods to assess its biological activity and employed to evaluate REST expression in cytological samples and cervical tissues. The anti-REST *scFv* represents an innovative detection tool that retains the antigen recognition of the parental IgM while overcoming its size limitation, enabling tissue penetration and detection of REST in cervical samples. Its application facilitates the identification of REST in cervical samples, reinforcing REST’s potential as a diagnostic biomarker for CC and CIN.

## 1. Introduction

CC is the fourth most common type of cancer among women worldwide until 2020 [1]. Despite its multifactorial development, infection with the high-risk human papillomavirus (HR-HPV) is the main associated factor due to its high persistence and viral integration. Infection with HR-HPV promotes the development of premalignant lesions, named as squamous intraepithelial lesions (SILs) or CIN [2]. To avoid the development of CC, it is important to perform an early diagnostic test, commonly the Pap smear, and confirm the diagnosis with histopathology and colposcopy [3]. In addition to the potential utility of the previously described methods for the diagnosis of premalignant lesions, it has been reported that cytological and histological methods are prone to error due to incorrect lesion classification, inter-observer variability, and subjective interpretation [4]. It is necessary to use biomarkers for the improvement of the diagnosis of premalignant lesions of the cervix. Recently, we proposed REST (RE1-Silencing Transcription factor) as a new biomarker to assist in the diagnosis of premalignant lesions and CC [5].

REST is a nine-zinc finger protein that binds to the Repressor Element-1 (RE-1) to silence hundreds of genes. Its primary role is the negative regulation of neurogenesis [6]. Additionally, REST regulates the expression of non-neuronal genes involved in various processes such as motility, angiogenesis, apoptosis, cellular division, and protein synthesis. It is considered a tumor suppressor gene [7], and the loss of its nuclear expression has been reported in several types of cancer, including prostate [8], breast [9], and CC [5]. In particular, in CC, negative nuclear expression of REST has been observed in 76.2% of cases with CIN II/III and 72.4% of cases with invasive squamous cell carcinoma. It has been proposed that REST expression could serve as a potential diagnostic or prognostic biomarker [5]. However, despite the potential of REST as a biomarker, there are currently only a few commercially available antibodies suitable for detecting REST in biological samples. In 2019, our research group developed an IgM monoclonal antibody specific for detecting REST in liquid cytology samples from women with SIL. Unfortunately, this IgM antibody was unable to detect the nuclear expression of REST in tissues from women with CIN I, likely due to its large size (900 kDa) and low penetrability [10]. For this reason, the purpose of this work was to design a smaller anti-REST antibody, but with the ability to recognize REST in its native form in cervical tissue-derived cells, based on the IgM monoclonal antibody.

Several biotechnological strategies have been developed to reduce antibody size while preserving their binding specificity. One of the most widely used approaches is the generation of *scFv*, compact functional units (~28 kDa) composed of the variable domains of both heavy (VH) and light (VL) chains connected by a flexible glycine–serine linker [11]. To our knowledge, applications of these technologies for detecting biomarkers in premalignant and malignant cervical lesions have not yet been reported. This study aims to develop a *scFv* anti-REST, derived from a previously generated IgM-producing hybridoma, capable of recognizing REST in its native nuclear form in cervical samples. This approach seeks to overcome the limitations of the high-molecular-weight IgM antibody for detecting REST in premalignant lesions and invasive cervical carcinoma. This work contributes to expanding the antibody engineering field applied to gynecologic oncology, offering an innovative approach to improve the detection of biomarkers in premalignant lesions and CC.

## 2. Results

### 2.1. scFv Construction

**Figure 1:**
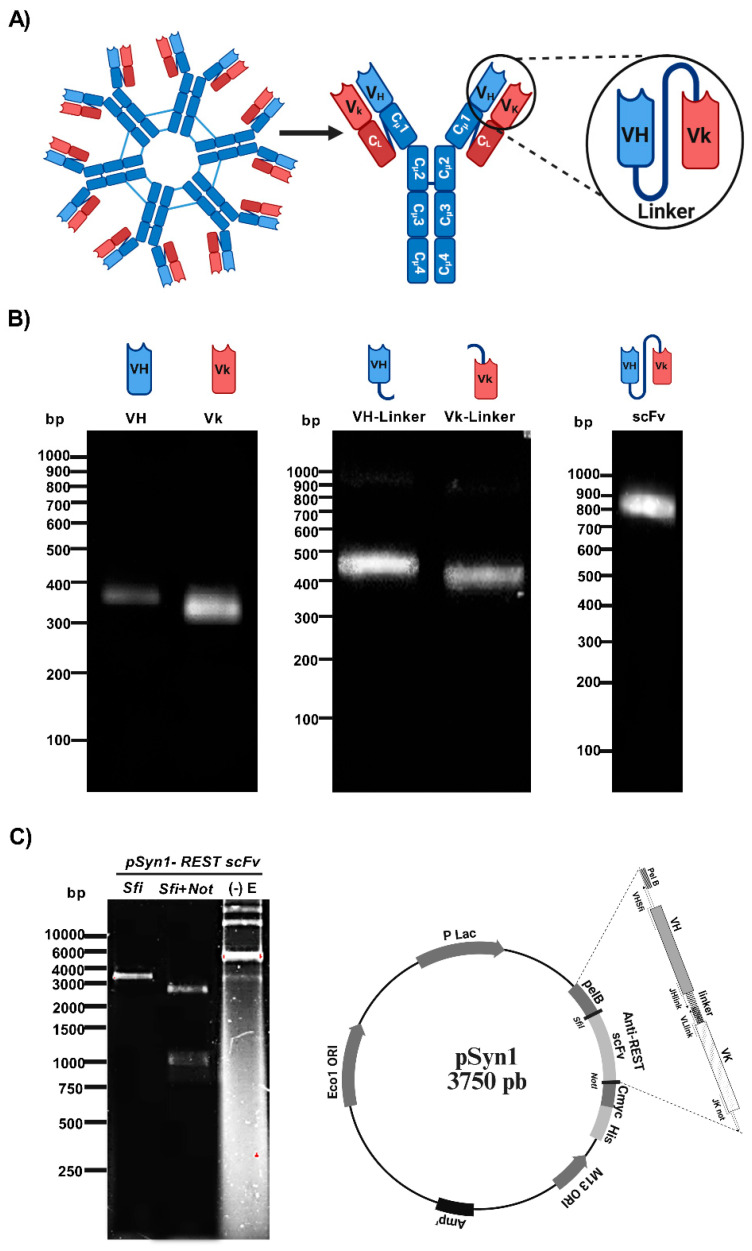
Design of the anti-REST *scFv*. (**A**) Schematic representation of the *scFv* derived from a previously obtained anti-REST IgM monoclonal antibody. The variable heavy (VH) and light (Vk) domains were linked by a flexible peptide linker to generate a single-chain format. (**B**) Electrophoresis showing the PCR products derived from the VH (340 bp) and Vκ (320 bp) domains of the hybridoma 5G2. Each VH and Vκ domain included the (Gly_4_S)_3_ linker, which possesses restriction sites for *SfiI* and *NotI*. Finally, overlapping PCR was utilized to construct the REST *scFv* segment. All PCR reactions were conducted on 1.5% agarose gels stained with GelRed (Biotium Cat# 41003). (**C**) Restriction and vector map of the recombinant plasmid *pSyn1–REST scFv*. The agarose gel shows plasmid digestion with *SfiI* and *NotI* restriction enzymes and the undigested plasmid (-) E. Schematic map of *pSyn1–REST scFv*. It shows the zoomed linear representation of the inserted *scFv* sequence.

To obtain a *scFv* against REST, the variable domains VH and Vk from a previously generated anti-REST IgM monoclonal antibody were used as templates (Figure 1A). Subsequently, the VH and Vk regions were amplified by RT-PCR and PCR and assembled through overlapping PCR using the linker sequence. Distinct amplification products were obtained for VH (~340 bp) and Vk (~320 bp). Restriction sites (*SfiI* to VH and *NotI* to Vκ) and half of the connector sequence were incorporated into each VH and Vκ fragment by PCR. The assembled *scFv* (~800 bp) displayed the expected size, confirming successful generation of the complete coding sequence (Figure 1B). The resulting *scFv* sequence was cloned into the *pSyn1* expression vector under the control of the lac promoter. This vector also includes a C-myc epitope tag fused to the C-terminus of the *scFv*, enabling its detection in downstream analyses when secondary antibodies require an Fc-region target. Figure 1C shows the restriction analysis of the vector: single digestion with *SfiI*, double digestion with *SfiI* and *NotI*, and the undigested plasmid lacking the *scFv* insert. The schematic map illustrates the recombinant *pSyn1–REST scFv* construct (~3750 bp), including the inserted *scFv* sequence, with a zoomed view highlighting the fusion between the pelB signal peptide and the *scFv* coding region.

### 2.2. scFv Cloning and Expression

**Figure 2:**
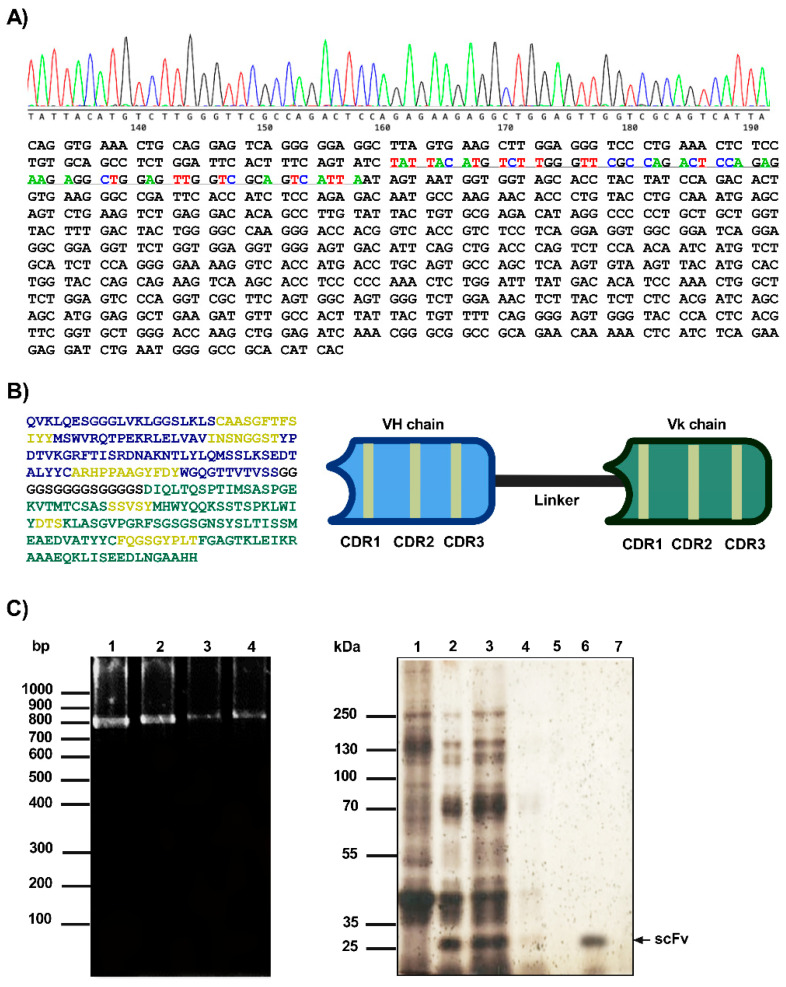
Sequence analysis, structural organization, and expression of the anti-REST *scFv*. (**A**) Nucleotide sequence of the cloned fragment encoding the anti-REST *scFv*. The chromatogram shows the quality of the sequencing reaction. (**B**) Predicted amino acid sequence of the *scFv* protein translated from the nucleotide sequence. The complementarity-determining regions (CDRs) are highlighted in yellow, the connecting linker in black, and the VH and Vk regions are indicated in blue and green, respectively. (**C**) (Left) Selection of the positive colonies for the *pSyn1-scFv*. PCR amplification of the sequence REST *scFv* from the selected colonies. Electrophoresis was performed in a 1.5% agarose gel stained with Gel Red (Biotium Cat# 41003) and a molecular weight of 100 bp (Thermo Scientific™ GeneRuler DNA Ladder Cat#SM0241). (Right) Expression and purification of the REST *scFv*. Lane 1: untransformed TG1 strain, lane 2: transformed TG1 strain, lane 3: induced TG1 strain with IPTG, lane 4–7: elution fractions of the *scFv*. SDS PAGE at 12% stained with silver.

The *pSyn1-scFv* construct was then used to transform the TG1 strain of *E. coli*. The colonies were cultivated for 16 H in YT2X medium with penicillin. We selected positive colonies containing the *pSyn1-scFv* insert by PCR (Figure 2C, Left), and the plasmids from these colonies were subsequently sequenced to confirm the anti-REST *scFv* sequence and the correct open reading frame. For the VH domain, the closest germline, diversity, and junction segments are identified as IGHV5-6-2*01F, IGHD2-14*01F, and IGHJ2*01F, respectively. In the VL domain, the germline and junction segments correspond to IGKV4*01F and IGKJ5*01F, respectively (Figure 2A). The deduced amino acid sequence confirmed the successful assembly of the anti-REST *scFv*, consisting of the VH domain, which corresponds to the N-terminal region, while the Vk domain occupies the C-terminal portion of the construct. Each domain contains the characteristic complementarity-determining regions (CDR1, CDR2, and CDR3), responsible for antigen recognition. The predicted organization illustrates the correct orientation and connectivity of both domains, preserving the canonical structure of a functional *scFv* (Figure 2B). Finally, the anti-REST *scFv* was recovered by periplasmic expression, processed, and purified using Ni-NTA columns (see Section 4). The purification process was confirmed through SDS-PAGE, where the REST *scFv* appeared in lane 6, corresponding to a molecular weight of 28.2 kDa (Figure 2C, Right).

### 2.3. Evaluation of the Biological Activity of the REST scFv

**Figure 3:**
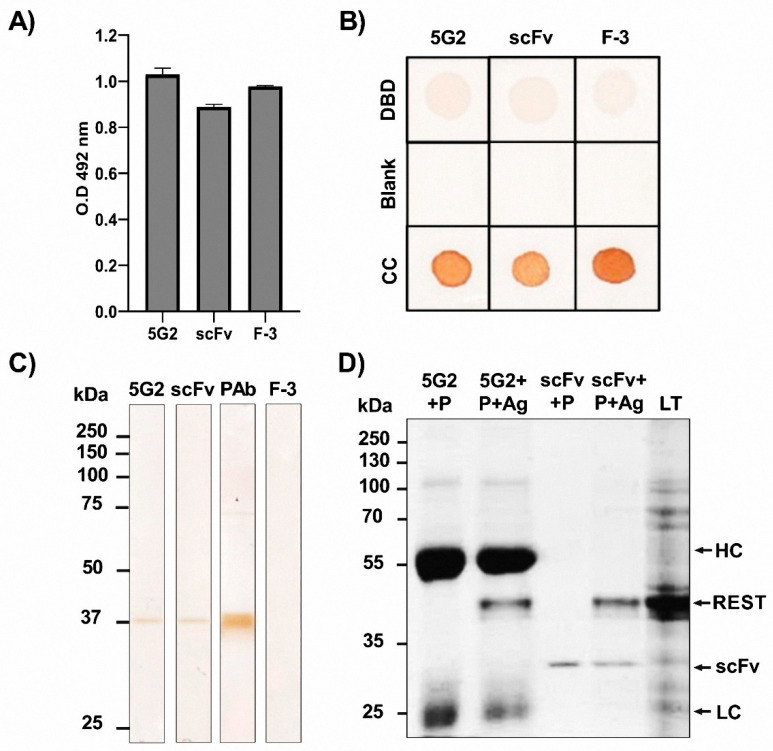
Biological activity of the *scFv*. (**A**) Indirect ELISA, (**B**) Dot blot, and (**C**) Western blot to test the biological capacity of the IgM antibody and the *scFv* against REST. Antigen: Recombinant DBD of REST to a final concentration of 0.1 µg/mL. Primary antibody: 5G2 IgM monoclonal antibody against REST and the REST *scFv*. Secondary antibody: anti-mouse IgG (H+L) coupled to HRP (Jackson ImmunoResearch Cat#115-035-003). In the *scFv* experiments, we used an anti-c-Myc antibody (Santa Cruz Biotechnology, cat# sc-40) as the intermediate antibody before the secondary antibody in all experiments. In the Western blot, we used an in-house rabbit polyclonal antibody against the DBD as a positive control. (**D**) Immunoprecipitation. SDS-PAGE at 14% stained with Coomassie blue. Lane 1: 5G2 + P (5G2 IgM anti-REST + L agarose beads). Lane 2: 5G2 + P + Ag (5G2 IgM anti-REST + L agarose beads antigen REST DBD). Lane 3: *scFv* + P (*scFv* anti-REST + L agarose beads). Lane 4: *scFv* + P + Ag (*scFv* anti-REST + L agarose beads + antigen REST DBD) and lane 5: LT (Total lysate of *E. coli* expressing the DBD of REST).

After purifying the *scFv* protein, we began characterizing its ability to recognize the cognate antigen, the DNA-binding domain (DBD) of REST, using various immunoassays. First, we confirmed the binding capacity of the REST *scFv* to the DBD using indirect ELISA and Dot Blot, demonstrating that it possessed similar recognition capabilities as the IgM parental hybridoma (5G2) and the commercial antibody (F-3) (Figure 3A,B). To evaluate the specific recognition of the complete protein at the expected molecular weight, we performed Western blot analysis. The results showed that the REST *scFv* and the hybridoma 5G2 had a lower recognition of the denatured recombinant DBD compared to the rabbit polyclonal antibody used as a positive control (Figure 3C). To further investigate, we conducted immunoprecipitation using the 5G2 IgM antibody and the *scFv* coupled to L-protein, using a complete cell lysate derived from the TG1 *E. coli* strain where the recombinant DBD was expressed. We observed the heavy (~65 kDa) and light chains (~25 kDa) of IgM, the *scFv* (~28 kDa), and the recombinant DBD of REST (~38 kDa) at their expected molecular weights. These findings provide evidence of the IgM and the *scFv’s* capacity to recognize the target protein from a complex sample like the total lysate (Figure 3D).

### 2.4. Utility of the scFv in the Detection of Squamous Intraepithelial Lesions

**Figure 4:**
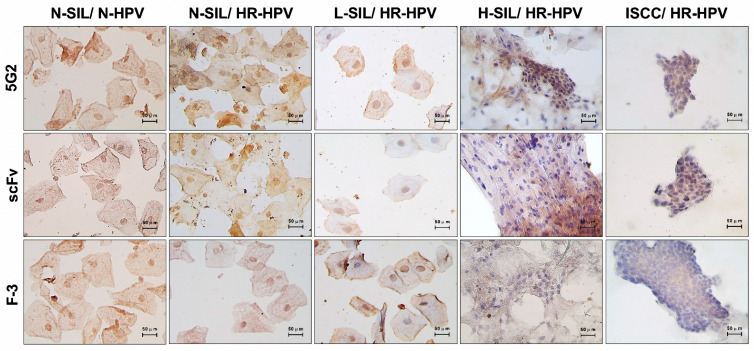
REST expression in liquid cytologies derived from a woman diagnosed with SIL and ISCC. Expression of REST by using the 5G2 IgM, the *scFv*, and the F-3 commercial IgG antibody by the immunocytochemistry technique using biotin–streptavidin–peroxidase. The reaction was developed with hydrogen peroxide and diaminobenzidine (DAB); Mayer’s hematoxylin was used as counterstaining. Samples were visualized at 40×.

To reduce the time required for immunocytochemistry and immunohistochemistry, we linked *scFv* to biotin (Supplementary Figure S1). This allowed us to employ a biotin–streptavidin–peroxidase technique. We conducted immunocytochemistry on 125 cytological samples obtained from women diagnosed with non-squamous intraepithelial lesion (N-SIL) and no HPV (n = 25), N-SIL and HR-HPV (n = 25), L-SIL and HR-HPV (n = 25), high-grade H-SIL and HR-HPV (n = 25), and invasive squamous cell carcinoma (ISCC) (n = 25). The clinical characteristics of the studied population are detailed in Supplementary Table S1. We analyzed the expression levels of REST, categorizing them as negative, low, mild, or intense, as well as its localization, which could be negative, in the nucleus, cytoplasm, or both. This analysis was conducted using three different antibodies: the parental 5G2 IgM, a commercial antibody (F-3), and the REST *scFv* (Figure 4).

During the analysis of the samples derived from woman without SIL we observed that independently of the infection by the HR-HPV, 100% of the samples express REST in the 96% of the cells in the nucleus and cytoplasm with 5G2 antibody and the 100% shows expressing of REST in nucleus and cytoplasm with the *scFv* and F-3 antibody, mostly cells with moderate to intense staining independently of the antibody used. In samples derived from patients with L-SIL we observed the loss of the nuclear expression of REST in comparison to the samples without SIL, in this group around 84% of the cells show a mild to moderate expression in the nucleus, and found that around 16% of the samples were negative by using the 5G2 antibody, 4% with the *scFv* and the 8% with the F-3 antibody. The expression was negative in the nucleus in 100% of the samples with H-SIL and ISCC independently of the type of antibody used (Table 1).

**Figure 5:**
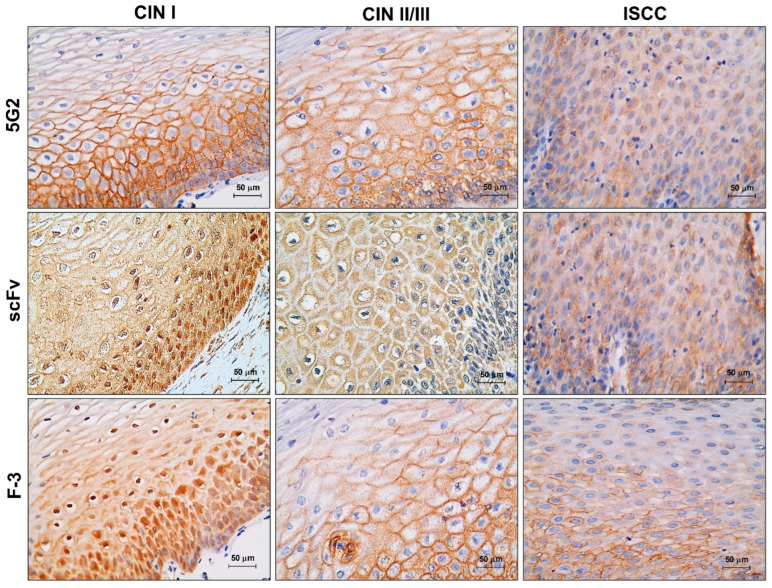
Expression of REST in tissues derived from women diagnosed with CIN and ISCC. REST expression was assessed using the 5G2 IgM, the *scFv*, and the F-3 commercial IgG antibody through immunohistochemistry techniques. The staining was developed using hydrogen peroxide and DAB, with Mayer’s hematoxylin used as a counterstain. Samples were visualized at 40× magnification.

The gold standard for diagnosing CIN is the histopathological analysis, which involves staining with hematoxylin and eosin and immunohistochemistry of cellular biomarkers. During the validation of the anti-REST IgM, the antibody failed to show nuclear expression of REST and instead observed membranous expression in CIN I with HR-HPV infection, although the same group of samples showed a nuclear pattern with the F-3 IgG commercial antibody. While the REST *scFv* managed to show the same expression pattern as the commercial antibody. In the CIN-II/III and ISCC, we observed cytoplasmic expression, independent of the type of antibody (Figure 5). To further support these observations, Supplementary Figure S2 includes immunohistochemistry of mouse breast tissue and brain, two tissues in which REST expression has been previously described, as well as a primary mouse breast tumor, which exhibited a staining pattern similar to that observed in high-grade cervical lesions and invasive carcinoma. These tissues served as additional biological controls to validate *scFv* reactivity. Moreover, negative controls in which the primary antibody was omitted were incorporated for human cervical tissue, confirming the specificity of the staining and excluding nonspecific background (Supplementary Figure S3).

In the CIN I group, both the *scFv* and F-3 antibodies demonstrated the expression of REST in the nucleus and cytoplasm in 100% of the tissues, with over two-thirds of the epithelium exhibiting this distribution. In contrast, the 5G2 antibody showed this expression pattern in only 12% of the tissues. In the CIN II/II group, regardless of the antibody used, we found that REST nuclear expression was completely lost in 100% of the tissues throughout the entire epithelial thickness, with its expression shifting to the cytoplasm. Finally, in the ISCC group, REST was entirely negative in the nucleus in 100% of cases, irrespective of the antibody used (Table 2).

## 3. Discussion

The successful amplification and assembly of the VH and Vk domains derived from the anti-REST IgM monoclonal antibody confirmed the feasibility of generating a recombinant *scFv* directed against REST. The fragment sizes obtained (~340 bp for VH and ~320 bp for Vκ) match the expected lengths for murine immunoglobulin variable domains, supporting both the specificity of the designed primers and the efficiency of the amplification conditions. Beyond confirming technical feasibility, these findings highlight that antibody miniaturization is an effective strategy to overcome the limitations of the parental IgM, whose large molecular size likely restricted tissue penetration. In contrast, the engineered *scFv* preserved REST specificity and enabled the detection of its native nuclear form in tissue samples, underscoring that reducing antibody size can enhance accessibility without compromising antigen recognition [12].

The incorporation of *SfiI* and *NotI* restriction sites, together with the (Gly_4_Ser)_3_ linker, enabled the precise assembly of the VH and Vκ fragments by overlap extension PCR, yielding the ~800 bp REST *scFv* sequence. The (Gly_4_Ser)_3_ linker is a well-established sequence that preserves flexibility between the VH and VL domains, facilitating proper folding and antigen recognition [13,14]. Compared with more complex assembly methods such as Gibson or In-Fusion cloning [15,16], overlap PCR remains a reliable and cost-effective alternative for constructing antibody fragments of moderate length. Its efficiency for *scFv* generation has been validated in multiple studies, and the results presented here are consistent with those reports [17,18]. This supports the suitability of the chosen strategy for producing a stable recombinant antibody sequence ready for subsequent cloning and expression.

The cloning of the REST *scFv* sequence into the *pSyn1* expression vector and its transformation into *E. coli* TG1 confirms that the molecular strategy employed was effective for recombinant antibody production. The detection of positive colonies by PCR and subsequent sequence verification ensured the correct assembly of the construct, confirming the presence and orientation of the insert as well as the integrity of the CDRs and the identification of germline origins—IGHV5-6-2*01F, IGHD2-14*01F, and IGHJ2*01F for the VH region, and IGKV4*01F and IGKJ5*01F for the VL region—suggests that the *scFv* retained native murine antibody characteristics. This finding supports the genetic fidelity of the amplified and cloned variable domains, indicating that no recombination or mutation events disrupted their natural configuration during amplification or ligation [19]. Overall, these findings confirm that the combination of the *pSyn1* vector system and periplasmic expression in *E. coli* is an effective approach for producing soluble, functional *scFv*. This is properly expressed and folded in the oxidizing periplasmic environment, which is favorable for disulfide bond formation, an essential feature of antibody structure [20]. The preservation of native CDRs and expected molecular weight strongly supports the structural integrity of the recombinant REST *scFv*, establishing a foundation for subsequent antigen-binding and functional characterization assays.

To evaluate the biological activity of the *scFv*, we employed several immunological techniques, including Western blotting, indirect ELISA, Dot blot, and immunoprecipitation. Through indirect ELISA and Dot blot assays, we demonstrated that both the 5G2 antibody and the *scFv* can recognize the native protein. This capability makes them suitable for techniques that detect REST in its functional form, which is important for diagnostic imaging of biological samples or in vivo [21]. Once native recognition was confirmed, immunoprecipitation provided a more stringent evaluation of antigen binding under complex biochemical conditions. Importantly, the *scFv* was able to selectively isolate the REST DNA-binding domain from total lysates of recombinant bacteria expressing the antigen, demonstrating that it can identify its target even in the presence of abundant nonspecific proteins. This result not only confirmed the formation of a stable antibody–antigen complex at the expected molecular weight but also validated the functional specificity of the *scFv* in a physiologically relevant environment [22].

The Western blot results confirm that our *scFv* exhibits low efficacy in recognizing the denatured protein, similar to that of the complete IgM antibody. Despite its simplified structure, the preservation of the antigen-binding site enables the *scFv* to replicate the expression patterns of the complete antibody. The lower recognition of the denatured DBD in Western blot compared to native forms observed in other techniques suggests that the *scFv* primarily recognizes conformational epitopes rather than linear ones. This behavior is characteristic of antibodies derived from IgM clones, which frequently depend on three-dimensional antigenic determinants for binding [23]. Conversely, the commercial F-3 antibody also failed to detect the recombinant REST DBD. This result can be explained by the difference in antigenic regions targeted by each antibody. The F-3 antibody is directed against the N-terminal portion of the full-length REST protein (amino acids 1–290), whereas both the REST *scFv* and the 5G2 hybridoma were specifically designed to recognize DBD located between amino acids 153–466. Therefore, when only the DBD fragment was used as the antigen, the F-3 antibody failed to efficiently bind its corresponding epitope, resulting in no signal in Western blot analysis [24].

After testing the biological function of the *scFv*, we assessed its potential use in diagnostics. The biotin–streptavidin–peroxidase system in ICC allowed a consistent evaluation of REST expression and localization across all cytological samples. When comparing the performance of the 5G2 IgM, the commercial F-3 antibody, and the newly generated *scFv*, no differences were observed in their ability to detect REST across the diagnostic groups. This equivalence indicates that the *scFv* is fully comparable to conventional antibodies in sensitivity and specificity, despite its smaller size and recombinant nature [25]. Additionally, it has been suggested that the loss of nuclear REST expression and its accumulation in the cytoplasm, observed in H-SIL and ISCC, may be due to the presence of isoforms that lack biological function and the absence of the nuclear localization signal (NLS) residues 512–522, which are necessary for nuclear entry [26].

During the immunohistochemical evaluation of REST, we observed results similar to those previously reported in cytology samples. This confirms that REST maintains moderate expression in both the nucleus and the cytoplasm in cases of CIN I, which typically revert or progress to H-SIL in only 20% of instances [27]. In CIN II/III and ISCC, REST is negative in the nucleus. This is associated with a lack of repression of its target genes [28], which can lead to processes that enhance neoplastic activity, such as increased proliferation through its interaction with STMN1 [29], epithelial–mesenchymal transition via its interaction with CDH2 [30], changes in cellular phenotype due to the re-expression of neuronal genes like SCN1 or BDNF [31,32], and the survival of tumor cells through the expression of GRIA1 and GRIA2, which encode glutamate receptors involved in synaptic signaling [33]. As seen in other types of cancer, the low expression of REST in cervical tissues of patients with CIN II/III and ISCC is associated with increased cellular proliferation, inhibition of apoptosis, activation of oncogenic signaling pathways, epithelial–mesenchymal transition, and metastasis [34,35].

The comparison of antibodies revealed important differences in their capacity to identify REST. The parental 5G2 IgM failed to detect the expected nuclear localization in CIN I with HR-HPV infection, instead producing an atypical membranous and cytoplasmic pattern. In contrast, both the commercial F-3 IgG antibody and the REST *scFv* accurately reproduced the nuclear expression expected for early-grade lesions, demonstrating that the *scFv* maintains appropriate antigen recognition within tissue architecture. These findings also support the notion that the large size of IgM limits its penetration into tissue, which likely explains the unexpected staining pattern. In contrast, the markedly smaller *scFv* retains full binding capacity and accesses nuclear epitopes more efficiently, performing similarly to the commercial IgG antibody. By reducing molecular size, recombinant formats like *scFv* not only improve immunohistochemical detection but may also offer advantages for future diagnostic applications, including in vivo imaging, due to their faster tissue diffusion and clearance [36].

Using biotin to label *scFv* significantly enhances the performance of immunological techniques. Since *scFv* lacks the Fc domain, it is not typically recognized by conventional secondary antibodies, necessitating the use of intermediate antibodies such as C-myc or histidine tags. This requirement can increase both the time and cost of immunoassays [37]. To address these challenges, we optimized the IHC technique by conjugating the *scFv* with biotin. This approach not only reduced the incubation time but also improved the intracellular uptake of the *scFv*. Additionally, the biotin–streptavidin method boosted the signal by approximately eight times compared to the standard use of a secondary antibody [38].

REST detection was validated in cervical samples, showing consistent recognition by the recombinant *scFv*. However, we recognize that the inclusion of controls as neuronal or neuroectodermal tumor-derived cell lines, where REST expression is markedly higher, would further support the specificity of the *scFv*. Due to the limited availability of such tissues, they were not included in the present work. However, their incorporation in future studies is planned to strengthen the validation of REST detection in additional cellular contexts. Finally, the *scFv* anti-REST described here demonstrates that antibody engineering is an effective approach to overcome structural and functional limitations of traditional monoclonal antibodies.

## 4. Materials and Methods

### 4.1. Cell Culture of IgM-Producing Hybridomas

We defrosted the 5G2 hybridoma, which produces IgM antibodies against REST, as previously characterized by our research group [10]. The hybridomas were cultured in a 150 cm^2^ culture flask using DMEM (Dulbecco’s Modified Eagle Medium) supplemented with 10% fetal bovine serum (Gibco Cat. # 12491-015) in a 5% CO_2_ environment. Once the culture reached 80% confluence, we separated the culture supernatant and used the cells for RNA extraction.

### 4.2. RNA Extraction from Hybridomas

Total RNA extraction from the 5G2 cells was performed using TRIzol (Invitrogen, cat# 15596018) following the supplier’s protocol. First, we added 1 mL of TRIzol™ to the cell pellet and incubated the tube on ice for 10 min. Next, we added 100 µL of chloroform and incubated it on ice for 3 min. We then centrifuged the mixture at 10,000 rpm for 1 min. The supernatant was transferred to a new tube, and 250 µL of isopropanol was added. This mixture was centrifuged at 13,000 rpm for 15 min, after which the supernatant was carefully removed. The RNA was washed twice with 500 µL of 75% ethanol. Finally, the pellet was allowed to dry at room temperature and was then resuspended in 20 µL of RNase-free water before being stored at −20 °C until needed.

### 4.3. Construction of scFv

The extracted RNA from 5G2 cells was used as a template to obtain complementary DNA (cDNA). We utilized the AMV RT-PCR kit (New England Biolabs, Ipswich, MA, USA, Cat# M0277S) by mixing the following components: 4 µL of DNase-free distilled water, 1 µL of dNTP, 2 µL of dT oligos, and 3 µL of RNA. The mixture was incubated for 5 min at 65 °C. Then, we added 4 µL of buffer, 1 µL of ribonuclease inhibitor, and 1 µL of RT enzyme. Finally, the mixture was incubated at 42 °C for one hour and subsequently inactivated at 85 °C for 5 min. To amplify the VH and Vκ domains, we prepared a mixture containing 2.5 µL of 10× buffer, 1.5 µL of MgCl_2_, 1 picomolar (pM) of primers, 1 µL of dNTP, 1 U of DNA Taq polymerase (Thermo Scientific, Waltham, MA, USA, Cat# EP0401), and 15.75 µL of water. PCR was performed with the following specifications: 30 cycles at 95 °C for 1 min, 55 °C for 1 min, and 72 °C for 1 min. The initial denaturation was performed at 95 °C for 5 min, and the final elongation was conducted at 72 °C for 10 min. We used previously reported primers by Orlandi et al., to amplify the heavy and light chains of the antibody [12]: Heavy chain (Forward: 5′ TGA GGA GAC GGT GAC CGT GGT CCC TTG GCC 3′, Reverse: 5′ AGG TSM ARC TGC AGS AGT C 3′) and κ light chain (Forward: 5′ GTT TAG ATC TCC AGC TTG GTC C 3′, Reverse: 5′ GAC ATT CAG CTG ACC CAG TCT C 3′). PCR products were visualized on 1% agarose gels using 100 bp (Thermo Scientific, Waltham, MA, USA, GeneRuler DNA Ladder, Cat# SM0241) and 1 kb (Thermo Scientific™ GeneRuler DNA Ladder, Cat# SM0311) molecular weight markers. The PCR products were VH (340 bp) and Vκ (320 bp) and were purified using the GeneJet purification kit (Thermo Scientific, Cat# K0691). Each fragment VH an VL were reamplified to append a DNA segment encoding half of the peptide linker (Gly4S)_3_ and the restriction sites: The primers used for this were: VH-Linker (5′ CCA CCA GAA CCT CCG CCT CCT GAT CCG CCA CCT CC TGA GGA GAC GGT GAC CGT GGT CC 3′), Vk-Linker (5′ GGC GGA TCA GGA GGC GGA GGT TCT GGT GGA GGT GGG AGT GAC ATT CAG CTG ACC CAG TCT C 3′), VH-*SfiI* (5′ GTC CTC GCA ACT GCG GCC CAG CCG GCC ATG GCC CAG GTS MAR CTG CAG SAG TC 3′), and Vk-*Not I* (5′ GAG TCA TTC TCG ACT TGC GGC CGC CCG TTT GAT CTC CAG CTT GGT CC 3′. PCR products were purified and used in an overlapping PCR to complete the REST *scFv*. The PCR product was purified and cut with the enzymes *SfiI* and *NotI* (both from New England Biolabs, Ipswich, MA, USA, Cat# R0123S and Cat# R0189S), and ligated to the plasmid *pSyn1* previously cut with the same restriction enzymes using T4 ligase.

### 4.4. Cloning and Expression of scFv in a Bacterial System

Electrocompetent *Escherichia coli* TG1 bacteria were electroporated with recombinant *scFv-pSyn1* constructs and then recovered in SOC medium for one hour. The bacteria were subsequently cultured on YT2X agar supplemented with 2% glucose and 200 µg/mL ampicillin for 24 h at 37 °C. Positive colonies containing the construct were selected using PCR with primers that flank the insert site [17]. From the positive colonies for the insert, plasmid extraction and purification were performed using the Zyppy™ Plasmid Miniprep Kit (Zymo Research, Irvine, CA, USA, Cat#D4036). The purified plasmids were sequenced at the DNA Synthesis and Sequencing Unit of the Biotechnology Institute at UNAM. The nucleotide sequences were compared with databases using the BLAST algorithm (https://blast.ncbi.nlm.nih.gov/Blast.cgi, Accessed: 24 April 2024) and the IMGT databases (https://www.imgt.org/, Accessed: 24 April 2024) to determine the corresponding germ lines of each domain.

For *scFv* expression, fresh transformed TG1 cells were cultured in 2.5 mL of YT2X under the same conditions for 12 h at 37 °C and 150 rpm as pre-inoculum. 500 mL of mediaYT2X. Glucose 0.1% and 200 µL Amp were inoculated and grown for 3 h. A final concentration of 1 mM IPTG (QIAGEN, Venlo, The Netherlands, Cat# 129921) was added to induce *scFv* expression for 6 h at 30 °C and 150 rpm. The culture medium was centrifuged at 4000 rpm for 15 min, and the supernatant was discarded. The pellet was then resuspended in 12.5 mL of lysis buffer (PPB: 200 mg/mL of sucrose, 1 mM EDTA, and 30 mM Tris HCl) and incubated on ice. After this, the sample was centrifuged at 4000 rpm for 15 min, and the supernatant was collected. To the resulting pellet, 12.5 mL of 5 mM MgSO_4_ was added. Following 20 min on ice, the tube was centrifuged at 14,000 rpm for 15 min. The supernatant obtained from this step was then mixed with the previously collected supernatant and dialyzed in PBS overnight. The molecular weight (28201.31 Da) and molar extinction coefficient of the *scFv* were calculated theoretically based on its sequence. Protein concentration was determined using UV spectrophotometry at λ = 280 nm, utilizing the calculated molar extinction coefficient of 47,580 M^−1^·cm^−1^.

### 4.5. Purification of scFv

The purification of *scFv* was carried out using affinity chromatography with Ni-NTA resin, following the instructions provided in the Ni-NTA Fast Start Kit from QIAGEN (Cat#30600). To begin, the column was packed with 1 mL of Ni-NTA resin and equilibrated with 5 mL of PBS containing 20 mM imidazole. Next, we added the total lysate to the column and performed washing steps using PBS with 35 mM imidazole to remove any proteins that did not bind to the resin. Finally, elution was conducted using PBS with 250 mM imidazole, and the sample was subsequently dialyzed against PBS. To enhance the immunocytochemical technique, the purified *scFv* was biotinylated.

### 4.6. Enzyme-Linked Immunosorbent Assay (ELISA)

The biological activity of the *scFv* was verified using an indirect ELISA. In this assay, recombinant REST served as the antigen, while purified mAbs and *scFvs* acted as the primary antibodies. An anti-c-Myc antibody (Santa Cruz Biotechnology, cat# sc-40) was utilized as the intermediate antibody, and an anti-mouse H+L antibody (Jackson ImmunoResearch, West Grove, Pennsylvania, USA, Cat# 115-035-003) was used as the secondary antibody. Microtiter plates (Sigma Aldrich, Burlington, MA, USA, Cat# CLS3590) were coated with the antigen by adding 100 μL of a 5 µg/mL protein solution in coating buffer (50 mM Na_2_CO_3_/NaHCO_3_, pH 9.6) and incubated overnight at 4 °C. The plates were then blocked for 40 min at 37 °C with 200 μL of a 5% skimmed milk solution in PBS-Tween (0.05%) before being incubated with the primary antibody for two hours at 37 °C. After washing, the plates received 100 μL of the anti-c-Myc antibody (diluted 1:2000) and were subsequently incubated with 100 μL of anti-mouse H+L conjugated to HRP (diluted 1:2000 in 0.05% PBS-Tween) for two hours at 37 °C. The enzymatic reaction was developed using O-phenylenediamine dihydrochloride (Sigma-Aldrich, Cat# P1526) and was terminated with 2 N H_2_SO_4_. Finally, the optical density was measured at 492 nm using a microplate reader (Tecan Sunrise, Tecan Group Ltd., Männedorf, Switzerland).

### 4.7. Dot Blots

The antigen was first immobilized on nitrocellulose membranes overnight. The next day, the membranes were blocked with 5% skim milk in 0.05% PBS-Tween for 40 min at room temperature while shaking. After washing, the membranes were incubated with mAbs and *scFvs* at final concentrations of 10 μg/mL and 75 ng/mL, respectively, for two hours at room temperature with constant agitation. This was followed by an additional incubation with anti-c-Myc in the case of the *scFv*. Finally, an HRP-conjugated anti-mouse H+L antibody was added at a dilution of 1:2000 in 0.05% PBS-Tween and incubated for two hours. The immunoreactivity was developed using 3,3′-diaminobenzidine (DAB, Sigma Aldrich Cat # D8001).

### 4.8. Immunoprecipitation

Pure *scFvs* (75 ng) and mAbs (10 µg) were incubated with 5 µL of protein L-agarose beads (sc-2336, Santa Cruz Biotechnology) at room temperature with shaking for 3 h. The beads were then washed three times with PBS. Next, 100 µg of lysate containing the recombinant REST DNA-binding domain was added to the antibody-bead complexes and incubated for an additional three hours with constant agitation. The complexes were washed as previously described. Immunoprecipitated REST was analyzed using 10% SDS-PAGE and stained with Coomassie blue, following the manufacturer’s instructions.

### 4.9. Electrophoresis and Western Blot

For the Western Blot, 100 µg of bacterial lysate containing recombinant REST DBD was denatured in loading buffer at 98 °C for 10 min (Biorad Cat # 1610747). The sample was then separated by 12% sodium dodecyl sulfate polyacrylamide gel electrophoresis (SDS-PAGE) and transferred to a nitrocellulose membrane (Biorad Cat # 1620115). The membrane was blocked with 5% skim milk in PBS-Tween 0.05% for 40 min at room temperature with shaking. After washing, the membrane was incubated with the following antibodies at room temperature with constant agitation: mAbs at a final concentration of 10 µg/mL, *scFvs* at 75 ng/mL, and rabbit polyclonal antibody anti-REST at 5 µg/mL. For *scFvs*, anti-Cmyc was applied at a dilution of 1:2000, followed by incubation with an HRP-conjugated anti-mouse antibody (dilution 1:2000) in PBS-Tween 0.05% for two hours. The immunoreactivity was developed using 3,3′-diaminobenzidine (DAB, Sigma-Aldrich Cat # D8001).

### 4.10. Sample Collection

All samples were obtained from the Cytopathology and Histochemistry Research Laboratory Biobank. A total of 60 histological samples were collected from patients diagnosed with CIN: 25 samples with CIN I, 25 samples with CIN II/III, and 10 samples with ISCC. These samples were collected from the lesion site through guided colposcopy by a certified pathologist, who conducted the histological analysis. Additionally, we analyzed 125 cervical cytology samples, which included N-SIL, L-SIL, H-SIL, and ISCC with HR-HPV infection. These samples were previously classified according to the Bethesda system. Exo-endocervical exfoliated cell samples were collected by sampling the ectocervix with an Ayre spatula. Samples from the endocervix were obtained using a cytobrush to ensure that cytological material was gathered from the squamous–columnar transformation zone. The cytological samples were preserved using a preservative solution (liquid-PREPTM, LGM International Inc., Melbourne, FL, USA) for at least one hour before processing. An aliquot of approximately 50 μL was placed on a clean glass microscope slide and fixed with 95% ethanol. These fixed specimens were then used for immunocytochemistry.

### 4.11. Biotinylation of scFv

One milligram of *scFv* was placed in a 0.1 M carbonate buffer at pH 8 and dialyzed overnight. The following day, we added 200 µL of biotin-N-hydroxysuccinimide (Sigma Aldrich, Cat# 0035013720) and incubated the mixture for 4 h at room temperature in the dark. To remove any unbound biotin, the solution was dialyzed in PBS at pH 7.2 overnight. The conjugated *scFv* was then stored in the dark at −20 °C until needed.

### 4.12. Immunocytochemistry and Immunohistochemistry

The immunocytochemistry and immunohistochemistry procedures were carried out using the streptavidin–biotin–peroxidase-based method, specifically employing the Cytoscan HRP/DAB cell detection system (Cell Marque Corporation, Hot Springs, AR, USA). The antibodies utilized in this study included biotinylated *scFv*, 5G2 mAb, and the commercially available anti-REST (F-3, Santa Cruz Biotechnology, Santa Cruz, CA, USA, Cat# sc-374611). The cytology slides underwent antigen retrieval using ImmunoDNA Retriever Citrate (Bio SB, Santa Barbara, CA, USA) for 15 min at 120 °C. To inhibit endogenous peroxidase activity, the slides were incubated with 3% hydrogen peroxide for 30 min. Following this, samples were incubated with the primary antibodies: 50 ng/mL for the biotinylated *scFv*, 10 µg/mL for 5G2, and a dilution of 1:100 for the F-3 commercial antibody, for a duration of 2 h. After the incubation, exogenous biotin and streptavidin–peroxidase were added for an additional 30 min. Finally, the reaction was developed using the chromogen DAB for 1 min and then counterstained with Mayer’s hematoxylin. As a negative control, the primary antibody was omitted.

### 4.13. Statistical Analysis

All statistical analyses were conducted using GraphPad Prism v. 8.0.1 and STATA v. 13. A *p*-value of less than 0.05 was deemed statistically significant.

## 5. Conclusions

The development and characterization of a recombinant *scFv* derived from the IgM 5G2 antibody improved the immunohistochemical detection of REST, particularly in identifying the nuclear expression of the protein, which was not feasible with the complete antibody. The effectiveness of the *scFv* was enhanced by linking it to biotin and utilizing it in a biotin–streptavidin system. This approach increased the detection capabilities in biological samples by reducing incubation times and enhancing intracellular detection. These advancements emphasize the value of recombinant antibodies as accessible and adaptable tools in resource-limited settings, such as developing countries. Although they strengthen the evidence supporting REST as a potentially informative marker in cervical lesions, current data do not establish it as a definitive or independent biomarker for the diagnosis of premalignant or malignant cervical disease. Rather, REST should be viewed as a complementary marker that may enhance diagnostic or prognostic evaluations when used alongside established clinical and pathological indicators.

## Figures and Tables

**Table 1:** REST expression in cervical cytology samples by using monoclonal antibodies and the *scFv*.

Evaluation Criteria	Non SIL/Non HPV n = 25 (%)	Non SIL/HPV-HR n = 25 (%)	L-SIL/HPV-HR n = 25 (%)	H-SIL/HPV-HR n = 25 (%)	ISCC n = 25 (%)	*p* *
**5G2**						
**Expression level**						
Negative	0	0	4 (16)	0	25 (100)	
Mild	1 (4)	3 (12)	5 (20)	16 (64)	0	<0.001
Moderate	4 (16)	19 (76)	16 (64)	9 (36)	0	
Intense	20 (80)	3 (12)	0	0	0	
**Location**						
Negative	0	0	4 (16)	0	25 (100)	
Nucleus	1 (4)	2 (8)	0	0	0	
Cytoplasm	0	0	0	25 (100)	0	<0.001
Nucleus–Cytoplasm	24 (96)	23 (92)	21 (84)	0	0	
***ScFv***						
**Expression level**						
Negative	0	0	1 (4)	0	15 (60)	
Mild	0	0	0	20 (80)	10 (40)	<0.001
Moderate	2 (8)	22 (88)	24 (96)	5 (20)	0	
Intense	23 (92)	3 (12)	0	0	0	
**Location**						
Negative	0	0	1 (4)	0	15 (60)	
Nucleus	0	0	0	0	0	
Cytoplasm	0	0	0	25 (100)	10 (40)	<0.001
Nucleus–Cytoplasm	25 (100)	25 (100)	24 (96)	0	0	
**F-3**						
**Expression level**						
Negative	0	0	2 (8)	0	12 (48)	
Mild	0	4 (16)	10 (40)	18 (72)	13 (52)	<0.001
Moderate	4 (16)	20 (80)	13 (52)	7 (28)	0	
Intense	21 (84)	1 (4)	0	0	0	
**Location**						
Negative	0	0	2 (8)	0	12 (48)	
Nucleus	0	0	0	0	0	
Cytoplasm	0	0	0	25 (100)	13 (52)	<0.001
Nucleus–Cytoplasm	25 (100)	25 (100)	23 (92)	0	0	

SIL: Squamous Intraepithelial Lesion; Non SIL: Without SIL; L-SIL: Low-grade SIL; H-SIL: High-grade SIL; ISCC: Invasive Squamous Cell Carcinoma; HPV: Human Papillomavirus; HR: High Risk. * X^2^ *p* < 0.05.

**Table 2:** Evaluation of REST expression in tissue samples diagnosed with CIN and ISCC with the commercial antibody, 5G2, and *scFv*.

Evaluation Criteria	CIN I n = 25 (%)	CIN II/III n = 25 (%)	ISCC n = 10 (%)	*p* *
**5G2**				
**Expression level**				
0	0	7 (28)	0	
1+	0	0	0	<0.001
2+	4 (16)	0	0	
3+	21 (84)	18 (72)	0	
Tumor nests in the stroma **Location**			10 (100)	
Negative	0	7 (28)		
Nucleus	0	0	0	<0.001
Cytoplasm	22 (88)	18 (72)	10 (100)	
Nucleus–Cytoplasm	3 (12)	0	0	
***scFv***				
**Expression level**				
0	0	0	0	
1+	0	0	0	<0.001
2+	5 (20)	0	0	
3+	20 (80)	25 (100)	0	
Tumor nests in the stroma **Location**			10 (100)	
Negative	0	0	0	
Nucleus	0	0	0	<0.001
Cytoplasm	0	25 (100)	10 (100)	
Nucleus–Cytoplasm	25 (100)	0	0	
**F-3**				
**Expression level**				
0	0	0	0	
1+	0	0	0	<0.001
2+	4 (16)	0	0	
3+	21 (84)	25 (100)	0	
Tumor nests in the stroma **Location**			10 (100)	
Negative	0	0	0	
Nucleus	0	0	0	<0.001
Cytoplasm	0	25 (100)	10 (100)	
Nucleus–Cytoplasm	25 (100)	0	0	

CIN: cervical intraepithelial neoplasia grades I to III. ISCC: Invasive Squamous Cell Carcinoma. Epithelial distribution: 0 (negative); 1+ (lower one third of the epithelium); 2+ (lower two thirds of the epithelium); 3+ (more than two thirds up to total thickness of the epithelium) * X^2^ *p* < 0.05.

## Data Availability

Data are contained within the article or Supplementary Material.

## Supplementary Materials

The following supporting information can be downloaded at: https://www.mdpi.com/article/10.3390/ijms262411946/s1.
