# Supplementary material for: Generation of a scFv Derived from an IgM-Producing Hybridoma for the Detection of REST Expression in Premalignant Lesions and Invasive Squamous Cell Carcinoma
Source: Int J Mol Sci. 2025 Dec 11;26(24):11946. doi: 10.3390/ijms262411946 (PMC12732971; doi:10.3390/ijms262411946)
Supplement: Supplementary file 1 [file ijms-26-11946-s001.zip › ijms-3952096-supplementary.pdf]

Supplementary Materials

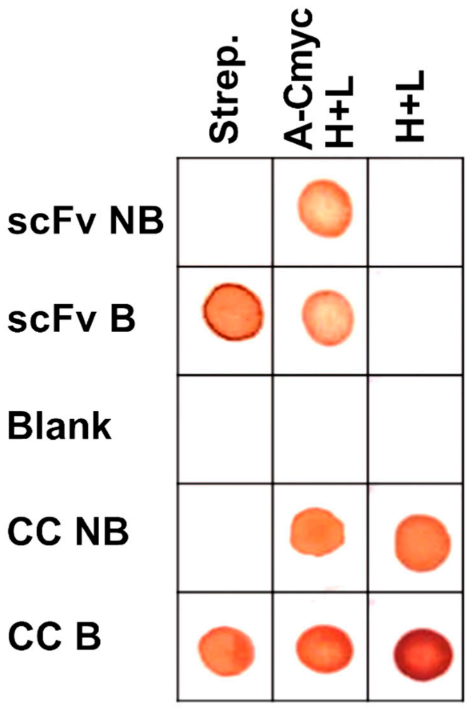

Supplementary Figure S1. Biotin Labeling of Anti-REST scFv Testing. Recombinant REST DNA-binding domain was used as the antigen. The study utilized both non-biotinylated single-chain variable fragments (scFv NB) and biotinylated single-chain variable fragments (scFv B) as primary antibodies. In the blank, the primary antibody was ommitted. For conjugate controls, non-biotinylated total mouse immunoglobulins (CC NB) and a biotin-coupled immunoglobulin (CC B) were included for direct binding of the secondary antibody. For reaction development, streptavidin coupled with horseradish peroxidase (Strep.) and a horseradish peroxidase-coupled anti-mouse heavy and light chains secondary antibody (H+L) were employed. Additionally, an anti-C-myc antibody was added to the H+L solution (A-Cmyc, H+L). The reaction was visualized using DAB and hydrogen peroxide.

Supplementary Table S1. Characteristics of the population.

Risk factor	Non SIL/ Non HPV n=25 (%)	Non SIL/ HPV-HR n=25 (%)	LSIL/ HPV-HR n=25 (%)	HSIL/ HPV-HR n=25 (%)	ISCC n=25 (%)	p^*
Age (years)						
18-30	2 (8)	5 (20)	12 (48)	8 (32)	0	
31-40	18 (72)	13 (52)	9 (36)	10 (40)	5 (20)	<0.001
41-50	5 (20)	5 (20)	4 (16)	2 (8)	10 (40)	
>50	0	2 (8)	0	5 (20)	10 (40)	
Sexual debut (years)						
<18						
18-25	8 (32)	7 (28)	16 (64)	17 (68)	18 (72)	
>25	15 (60)	15 (60)	9 (36)	8 (32)	6 (24)	0.008
	2 (8)	3 (12)	0	0	1 (4)	
Number of sex partners						
1	15 (60)	17 (68)	9 (36)	13 (52)	11 (44)	0.149
2-3	10 (40)	4 (16)	13 (52)	10 (40)	12 (48)	
>3	0	4 (16)	3 (12)	2 (8)	2 (8)	
Giving birth						
0	4 (16)	6 (24)	4 (16)	3 (12)	2 (8)	
1-3	18 (72)	15 (60)	14 (56)	12 (48)	12 (48)	0.190
>3	3 (12)	4 (16)	7 (28)	10 (40)	11 (44)	

SIL: Squamous Intraepithelial Lesion, LSIL: Low-grade SIL, HSIL: High-grade SIL, ISCC: Invasive Squamous Cell Carcinoma, HPV: Human Papillomavirus, HR: High Risk. *X^2 $p < 0.05$

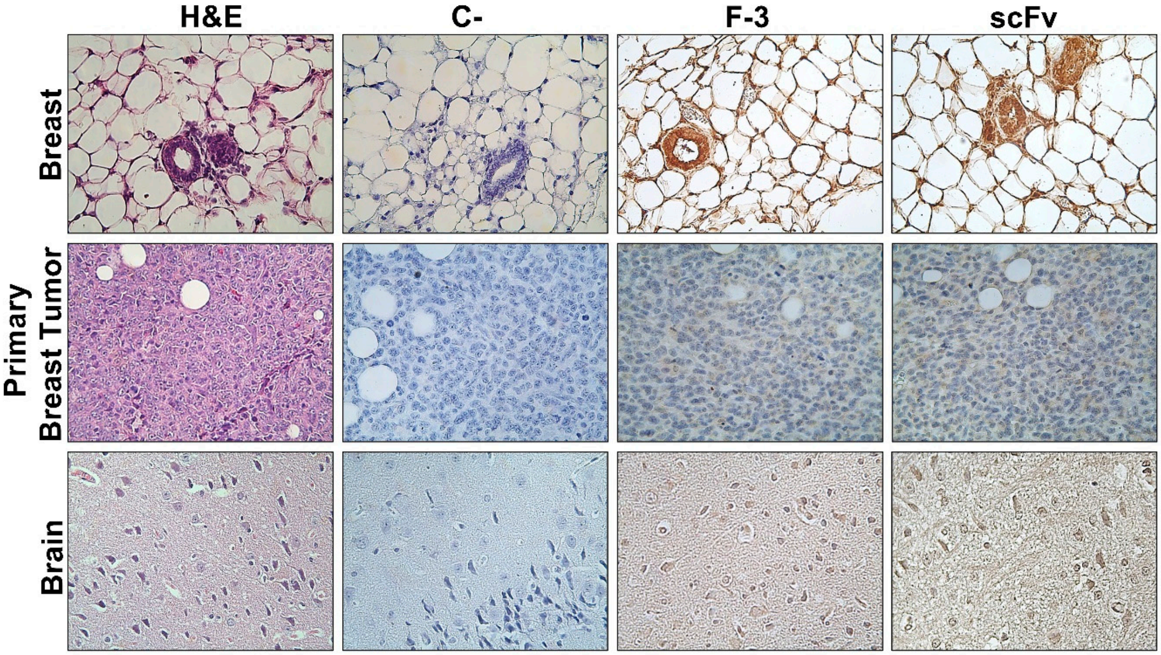

Supplementary Figure S2. Immunohistochemical detection of REST in mouse tissues using a biotin–streptavidin peroxidase system. REST expression was evaluated in mouse tissues; breast, primary breast tumor, and brain using a biotinylated anti-REST scFv and the commercial anti-REST antibody F-3 (Santa Cruz Biotechnology). Hematoxylin and eosin staining (H&E) and negative controls in which the primary antibody was omitted (C-) are shown for each tissue. Immunoreactivity was visualized with diaminobenzidine (DAB). All images were acquired at 40× magnification.

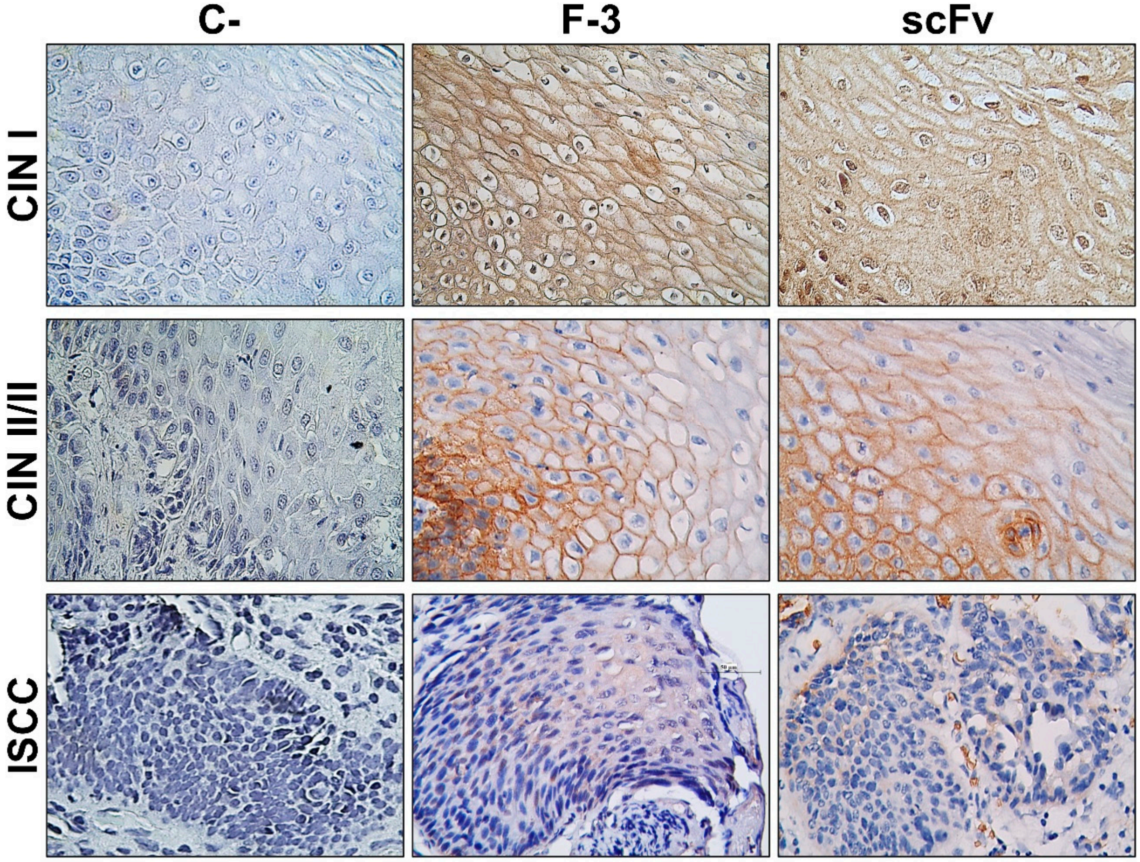

Supplementary Figure S3. Immunohistochemical detection of REST in cervical lesions using a streptavidin–peroxidase system. REST expression was evaluated in cervical intraepithelial neoplasia grade I (CIN I), grade II/III (CIN II/III), and invasive squamous cell carcinoma (ISCC) using the biotin–streptavidin peroxidase method. Staining was performed with the biotinylated anti-REST scFv and the commercial anti-REST antibody F-3 (Santa Cruz Biotechnology). Negative controls (C−) were included by omitting the primary antibody. All images were acquired at 40× magnification.
